# Development of pituitary dysfunction and destructive thyroiditis is associated with better survival in non-small cell lung cancer patients treated with programmed cell death-1 inhibitors: a prospective study with immortal time bias correction

**DOI:** 10.3389/fendo.2024.1490042

**Published:** 2024-11-07

**Authors:** Koji Suzuki, Tomoko Kobayashi, Tetsushi Izuchi, Koki Otake, Masahiko Ando, Tomoko Handa, Takashi Miyata, Mariko Sugiyama, Takeshi Onoue, Daisuke Hagiwara, Hidetaka Suga, Ryoichi Banno, Tetsunari Hase, Megumi Inoue, Makoto Ishii, Hiroshi Arima, Shintaro Iwama

**Affiliations:** ^1^ Department of Endocrinology and Diabetes, Nagoya University Graduate School of Medicine, Nagoya, Japan; ^2^ Center for Advanced Medicine and Clinical Research, Nagoya University Hospital, Nagoya, Japan; ^3^ Research Center of Health, Physical Fitness and Sports, Nagoya University, Nagoya, Japan; ^4^ Department of Respiratory Medicine, Nagoya University Graduate School of Medicine, Nagoya, Japan; ^5^ Department of Clinical Oncology and Chemotherapy, Nagoya University Hospital, Nagoya, Japan

**Keywords:** pituitary, destructive thyroiditis, PD-1, immune checkpoint inhibitors, immune-related adverse events

## Abstract

**Background:**

Immune-related adverse events (irAEs) are reported to be associated with better overall survival (OS) in non-small cell lung cancer (NSCLC) patients treated with immune checkpoint inhibitors. However, there may be a bias in that patients who develop irAEs must survive long enough to experience the irAEs, and no prospective studies adjusting for immortal time bias (ITB) have examined the relationship between OS and pituitary dysfunction or the two different types of thyroid dysfunction: destructive thyroiditis and hypothyroidism without prior thyrotoxicosis (isolated hypothyroidism).

**Methods:**

Patients with NSCLC who received nivolumab or pembrolizumab at Nagoya University Hospital between November 2, 2015 and February 1, 2023 were enrolled. Endocrine irAEs were prospectively assessed during scheduled evaluations of hormone levels. The association between irAE development and survival when considering ITB was examined by time-dependent Cox regression analysis.

**Results:**

Of the 194 patients included, 11 (5.7%), 10 (5.2%), and 5 (2.6%) developed pituitary dysfunction, destructive thyroiditis, and isolated hypothyroidism, respectively. The development of pituitary dysfunction (HR 0.36, 95% CI 0.13–0.98, p = 0.045) and destructive thyroiditis (HR 0.31, 95% CI 0.10–0.97, p = 0.044), but not isolated hypothyroidism (HR 1.15, 95% CI 0.42–3.20, p = 0.786), was significantly associated with longer OS.

**Conclusion:**

NSCLC patients developing pituitary dysfunction and destructive thyroiditis showed better OS even after adjusting for ITB, suggesting that these irAEs indicate a better prognosis.

## Introduction

Immune checkpoint inhibitors (ICIs) have shown striking efficacy against many types of cancer and currently play a crucial role in cancer therapy. However, despite their benefits, ICIs can trigger a diverse range of adverse events known as immune-related adverse events (irAEs), which occur in various organs including endocrine glands (1–4). Several studies have reported a relationship between the development of irAEs and better survival outcomes in patients treated with ICIs (5, 6). Recently, we reported that the development of pituitary and thyroid dysfunction after ICI treatment was associated with longer overall survival (OS) in non-small cell lung cancer (NSCLC) patients (7). However, this study may have been subjected to immortal time bias (ITB) because patients who develop irAEs must survive long enough to experience the irAEs. This bias may lead to overestimation of the survival benefits associated with irAEs, especially in the case of late-onset irAEs such as pituitary dysfunction (8). Using a time-dependent Cox model, several retrospective studies reported that the development of overall irAEs remained associated with improved OS in NSCLC patients even after taking into account ITB (9, 10). Furthermore, a subgroup analysis revealed associations of thyroid dysfunction and skin toxicity with longer OS (10). In addition, one prospective study in patients with concurrent NSCLC, renal cell carcinoma, and melanoma reported that thyroid dysfunction was associated with improved progression-free survival and OS when considering ITB (11). However, to our knowledge, no prospective studies have shown that the onset of pituitary dysfunction, which generally occurs several months after ICI initiation, has a survival benefit in the setting of ITB. Moreover, no prospective studies have examined the survival benefits of the two different types of thyroid dysfunction, destructive thyroiditis and hypothyroidism without prior thyrotoxicosis (isolated hypothyroidism), while taking into account ITB. In this prospective observational real-world study, we examined the relationship between the development of irAEs and survival in NSCLC patients treated with anti-programmed cell death-1 (PD-1) immunotherapy, taking into account ITB.

## Methods

### Study population

**Figure 1:**
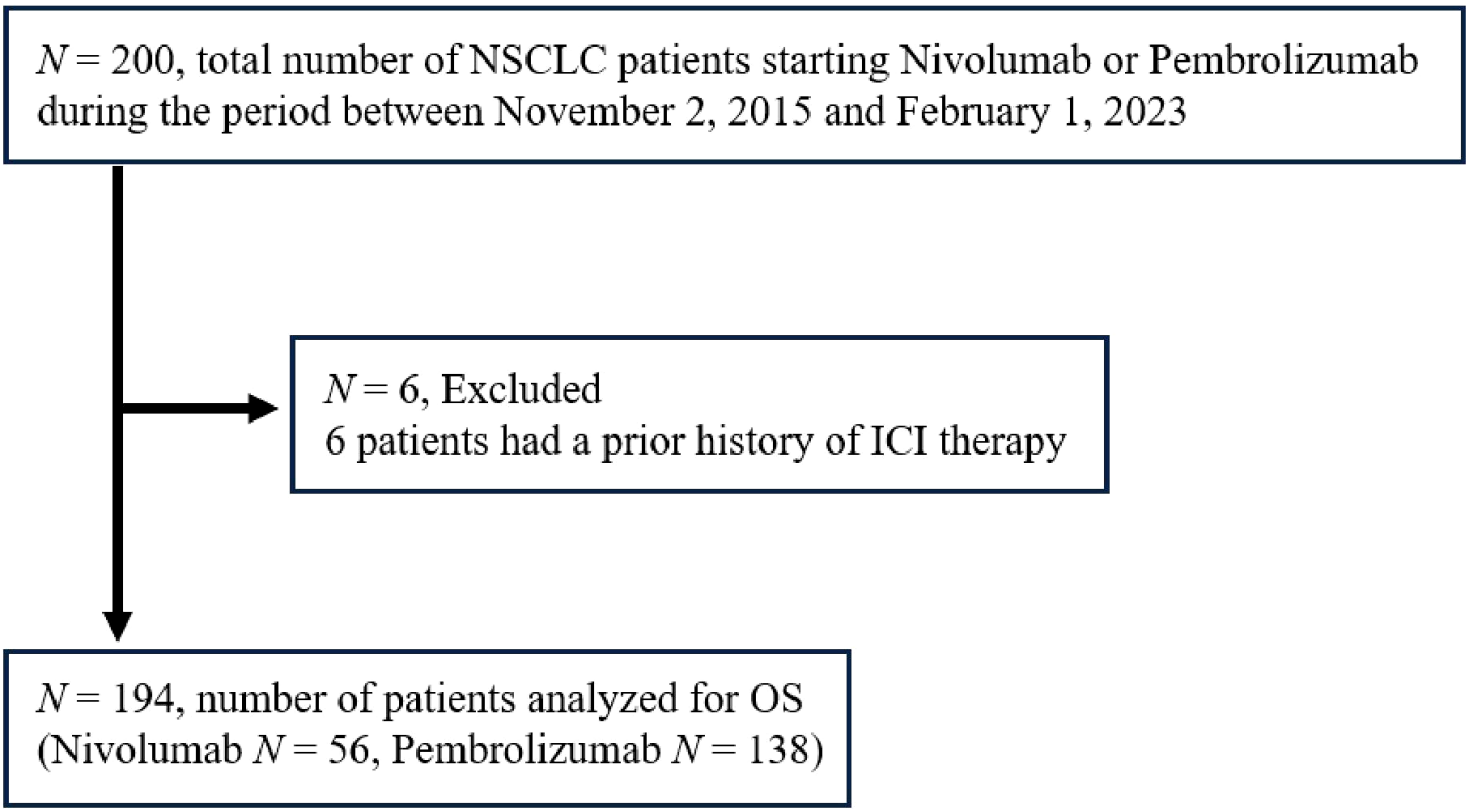
Enrollment of the study subjects. NSCLC, non-small cell lung cancer; ICI, immune checkpoint inhibitor; OS, overall survival.

In this prospective study analyzing endocrine irAEs in patients treated with ICIs (UMIN000019024), patients with NSCLC who had received at least one cycle of treatment with nivolumab or pembrolizumab at Nagoya University Hospital between November 2, 2015 and February 1, 2023 (**Figure 1**) were included. This study focused exclusively on patients treated with anti-PD-1 antibody therapy for the analyses, because the incidence of irAEs and the prognosis may vary depending on the class of ICIs. Patients with a history of any prior ICI treatment were excluded. This study was conducted following the ethical principles of the Declaration of Helsinki and was approved by the Ethics Committee of Nagoya University Hospital (Approval No. 2015-0273). Written informed consent was obtained from all patients prior to study enrollment.

### Data collection and definition of irAEs

Baseline clinical data, including age, sex, Eastern Cooperative Oncology Group Performance Status (ECOG-PS), and presence of distant metastasis (i.e. bone, liver, and brain), were collected through electronic medical records. To evaluate endocrine irAEs, the levels of adrenocorticotropic hormone, cortisol, and blood glucose were assessed at baseline and every 6 weeks after the initial ICI administration for 48 weeks, as described previously (12). Free triiodothyronine, free thyroxine, and thyroid-stimulating hormone (TSH) levels were assessed at baseline and 6, 12, 18, 24, 36, and 48 weeks after the first administration of ICIs based on a minor change to the study protocol. These measurements were also conducted when clinically needed. Each endocrine irAE was defined according to the Japan Endocrine Society clinical guidelines for endocrine irAEs (13). Thyroid dysfunction was classified into two types: 1) destructive thyroiditis (development of transient thyrotoxicosis in the absence of TSH receptor antibody (TRAb) positivity and 2) isolated hypothyroidism (development of hypothyroidism without preceding thyrotoxicosis). Non-endocrine irAEs were defined based on the judgment of attending physicians and/or clinical improvement after treatment with corticosteroids or other immunosuppressive agents. Non-endocrine irAEs were categorized based on the organ involved and were graded based on the National Cancer Institute Common Terminology Criteria for Adverse Events, version 5.0. Patients who developed multiple irAEs were assigned to the irAE group for analysis of the relationships between OS and each individual irAE. Patients were followed up until November 1, 2023.

### Statistical analysis

The study endpoint was OS, which was defined as the time from initial ICI treatment to death from any cause. To address ITB, univariate and multivariate Cox regression analyses were performed using each irAE as the time-dependent covariate to evaluate the correlation between OS and any irAE that occurred in at least five patients. In the analysis of overall irAEs, the time of onset was defined as the time from ICI initiation to the first irAE development. Other covariates included in the multivariate Cox regression analysis were age, sex, ECOG-PS, histology, and presence of metastasis. Continuous and categorical variables were compared between patients with and those without irAEs using the Mann–Whitney U test and Fisher exact test, respectively. All statistical tests were two sided, and significance was defined as a p value < 0.05. Statistical analyses were conducted using IBM SPSS Statistics, version 29.

## Results

### Patient characteristics

A total of 194 NSCLC patients treated with nivolumab or pembrolizumab were included in the analysis (**Figure 1**). The patient baseline characteristics are presented in **Table 1**. The median age at the initiation of ICI therapy was 70 years. Of the 194 patients, 75.3% were male, and 30.4% had squamous cell carcinoma. Regarding the ICI agent, 56 patients received nivolumab and 138 patients pembrolizumab (of whom 59 received it as combination therapy with chemotherapy [cisplatin, carboplatin, pemetrexed, or nab-paclitaxel]). The median follow-up period for the whole cohort was 410 (interquartile range [IQR] 179–882) days.

**Table 1:** Baseline patient characteristics and distribution of each irAE type.

Characteristic	No. (%) or median (IQR)
	All patients (n = 194)
Age, years	70 (61–74)
<75	156 (80.4%)
≥75	38 (19.6%)
Sex	
Female	48 (24.7%)
Male	146 (75.3%)
ECOG-PS	
≤1	181 (93.3%)
≥2	13 (6.7%)
Histology	
Non-squamous	135 (69.6%)
Squamous	59 (30.4%)
Metastasis	
No	105 (54.1%)
Yes	89 (45.9%)
Follow-up days	410 (179–882)
No. of irAEs	
1	53 (27.3%)
≥2	29 (14.9%)
irAEs	
Overall	82 (42.3%)
Pituitary dysfunction	11 (5.7%)
Thyroid dysfunction	15 (7.7%)
Isolated hypothyroidism	5 (2.6%)
Destructive thyroiditis	10 (5.2%)
Diabetes	3 (1.5%)
Skin toxicity	27 (13.9%)
Pneumonitis	24 (12.4%)
Hepatic toxicity	11 (5.7%)
Gastrointestinal toxicity	12 (6.2%)
Pancreatic toxicity	1 (0.5%)
Arthritis	3 (1.5%)
Neurologic toxicity	2 (1.0%)
Other	6 (3.1%)

irAE, immune-related adverse event; IQR, interquartile range; ECOG-PS, Eastern Cooperative Oncology Group performance status.

### irAE profiles

**Figure 2:**
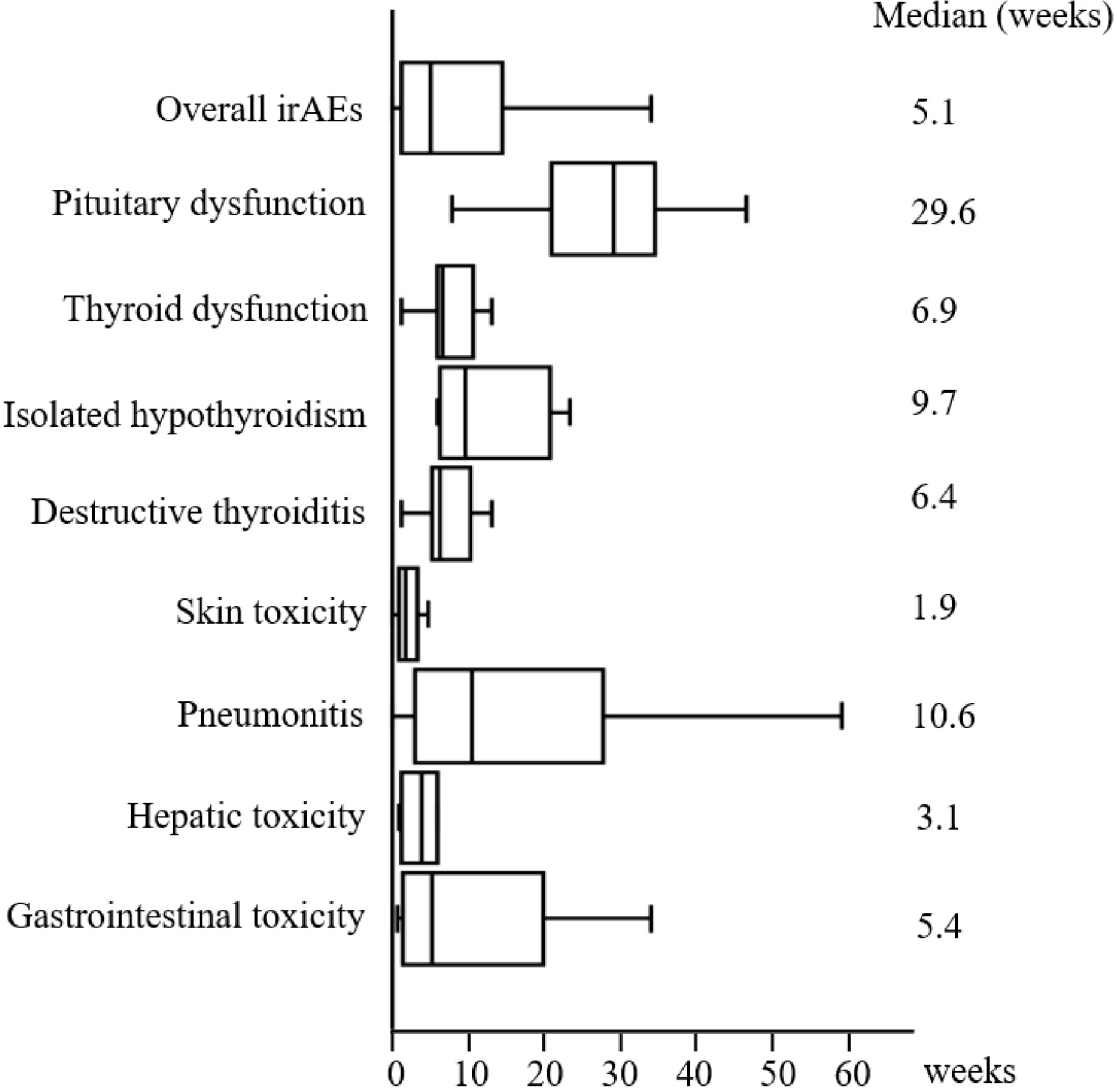
Time of irAE onset. The median time (in weeks) from the first administration of ICI therapy to the development of each irAE. The left, middle, and right lines of the boxes correspond to the 25^th^, 50^th^, and 75^th^ percentiles, respectively. The whiskers extend from the minimum to maximum times. irAE, immune-related adverse event.

During the follow-up period, 82 (42.3%) patients experienced 115 irAEs of all grades. The most common irAEs were skin toxicity (n = 27, 13.9%), pneumonitis (n = 24, 12.4%), thyroid dysfunction (n = 15, 7.7%), gastrointestinal toxicity (n = 12, 6.2%), hepatic toxicity (n = 11, 5.7%), and pituitary dysfunction (n = 11, 5.7%) (**Table 1**). Among the patients with thyroid dysfunction, 10 (5.2%) and 5 (2.6%) developed destructive thyroiditis and isolated hypothyroidism, respectively (**Table 1**). The median time of onset of each irAE type is shown in **Figure 2**. The median time to develop the first irAE was 5.1 (IQR 1.3–14.0) weeks. Pituitary dysfunction developed significantly later than did the other irAEs (median 29.6 [IQR 21.9–34.1] vs. 4.4 [IQR 1.9–10.9] weeks, p < 0.001). Of the 82 patients, 53 developed only one irAE, whereas 29 presented with two or more irAEs (**Table 1**). All cases of pituitary dysfunction were managed with hospitalization for treatment and pituitary function assessment and were classified as grades 3–5 (**Supplementary Table 1**). However, none of the patients with pituitary dysfunction were treated with high-dose glucocorticoids (**Supplementary Table 1**). Skin toxicity and thyroid dysfunction comprised mainly grades 1–2 irAEs, while hepatic toxicity, gastrointestinal toxicity, and pneumonitis tended to be more severe, with grades 3–5 irAEs accounting for 33.3–55.5% of cases (**Supplementary Table 1**). A substantial number of patients who developed severe irAEs discontinued ICI therapy and were treated with high-dose glucocorticoids (**Supplementary Table 1**). Of the 82 patients who developed any irAE, 31 were treated with systemic glucocorticoids. Among these 31 patients, 6 discontinued glucocorticoids, whereas the remaining 25 were still on glucocorticoids at the end of the study period.

### Univariate analysis of the association between irAE development and survival

To evaluate the association between irAE development and OS taking into account ITB, we performed time-dependent Cox proportional hazards regression analysis using irAEs as the time-dependent covariate. In addition, regarding thyroid dysfunction, subgroup analyses were performed separately for patients with isolated hypothyroidism versus destructive thyroiditis. The univariate analyses showed that ECOG-PS ≥ 2 (hazard ratios [HR] 7.38, 95% confidence interval [CI] 3.93–13.87, p < 0.001) and presence of metastasis (HR 1.61, 95% CI 1.15–2.26, p = 0.006) were significantly associated with shorter OS, whereas the developments of overall irAEs (HR 0.57, 95% CI 0.39–0.82, p = 0.003), pituitary dysfunction (HR 0.33, 95% CI 0.12–0.91, p = 0.031), destructive thyroiditis (HR 0.29, 95% CI 0.09–0.92, p = 0.036), and skin toxicity (HR 0.46, 95% CI 0.25–0.84, p = 0.011) were significantly associated with longer OS (**Table 2**). On the other hand, there was no significant association between OS and the development of overall thyroid dysfunction, isolated hypothyroidism, hepatic toxicity, gastrointestinal toxicity, or pneumonitis (**Table 2**).

**Table 2:** Time-dependent univariate Cox regression analysis of OS.

Characteristic	Univariate analysis	
	HR (95% CI)	p value
Age, years		
<75	1	
≥75	0.82 (0.54–1.26)	0.367
Sex		
Female	1	
Male	1.20 (0.80–1.79)	0.384
ECOG-PS		
≤1	1	
≥2	7.38 (3.93–13.87)	<0.001
Histology		
Non-squamous	1	
Squamous	1.12 (0.77–1.61)	0.557
Metastasis		
No	1	
Yes	1.61 (1.15–2.26)	0.006
irAEs		
Overall	0.57 (0.39–0.82)	0.003
Pituitary dysfunction	0.33 (0.12–0.91)	0.031
Thyroid dysfunction	0.50 (0.23–1.08)	0.076
Isolated hypothyroidism	1.14 (0.42–3.08)	0.802
Destructive thyroiditis	0.29 (0.09–0.92)	0.036
Skin toxicity	0.46 (0.25–0.84)	0.011
Pneumonitis	1.02 (0.58–1.78)	0.950
Hepatic toxicity	1.55 (0.76–3.18)	0.232
Gastrointestinal toxicity	1.16 (0.59–2.29)	0.669

OS, overall survival; HR, hazard ratio; CI, confidence interval; ECOG-PS, Eastern Cooperative Oncology Group performance status; irAE, immune-related adverse event.

Hazard ratio of the association between irAE and OS was calculated by time-dependent univariate Cox proportional hazards regression analysis.

### Multivariate analysis of the associations of pituitary dysfunction and destructive thyroiditis with OS

To identify the irAEs that are independent prognostic factors for OS, we performed time-dependent multivariate Cox regression analyses using irAEs as the time-dependent covariate, with adjustments for clinical variables (age, sex, ECOG-PS, histology, and metastasis). In addition to an ECOG-PS of 0–1 and absence of metastasis, pituitary dysfunction was independently associated with prolonged OS (HR 0.36, 95% CI 0.13–0.98, p = 0.045) (**Table 3**). There were no significant differences in the baseline characteristics (age, sex, ECOG-PS, histology, and metastasis) between patients who developed pituitary dysfunction and those who did not (**Supplementary Table 2**). Similarly, destructive thyroiditis was independently associated with prolonged OS (HR 0.31, 95% CI 0.10–0.97, p = 0.044) (**Table 4**). There were no significant differences in baseline characteristics between patients who developed destructive thyroiditis and those who did not (**Supplementary Table 3**). Overall irAEs (**Supplementary Table 4**) and skin toxicity (**Supplementary Table 5**) were also identified as independent prognostic factors for OS in the multivariate analyses. There were no significant differences in baseline characteristics between patients who developed skin toxicity and those who did not (**Supplementary Table 6**), whereas the prevalence of ECOG-PS ≥ 2 was significantly higher among patients who developed overall irAEs than those who did not (2/82 [2.4%] vs. 11/112 [9.8%], p = 0.046, **Supplementary Table 7**).

**Table 3:** Time-dependent multivariate Cox regression analysis of the association between pituitary dysfunction and OS.

Characteristic	Multivariate analysis	
	HR (95% CI)	p value
Age, years		
<75	1	
≥75	0.77 (0.49–1.22)	0.267
Sex		
Female	1	
Male	1.04 (0.68–1.59)	0.862
ECOG-PS		
≤1	1	
≥2	6.91 (3.62–13.18)	<0.001
Histology		
Non-squamous	1	
Squamous	1.18 (0.78–1.77)	0.438
Metastasis		
No	1	
Yes	1.52 (1.07–2.15)	0.019
Pituitary dysfunction		
No	1	
Yes	0.36 (0.13–0.98)	0.045

OS, overall survival; HR, hazard ratio; CI, confidence interval; ECOG-PS, Eastern Cooperative Oncology Group performance status.

**Table 4:** Time-dependent multivariate Cox regression analysis of the association between destructive thyroiditis and OS.

Characteristic	Multivariate analysis	
	HR (95% CI)	p value
Age, years		
<75	1	
≥75	0.76 (0.48–1.19)	0.223
Sex		
Female	1	
Male	1.01 (0.66–1.54)	0.979
ECOG-PS		
≤1	1	
≥2	6.77 (3.55–12.92)	<0.001
Histology		
Non-squamous	1	
Squamous	1.20 (0.80–1.81)	0.373
Metastasis		
No	1	
Yes	1.54 (1.09–2.18)	0.015
Destructive thyroiditis		
No	1	
Yes	0.31 (0.10–0.97)	0.044

OS, overall survival; HR, hazard ratio; CI, confidence interval; ECOG-PS, Eastern Cooperative Oncology Group performance status.

## Discussion

To our knowledge, this is the first prospective study to demonstrate that pituitary dysfunction and destructive thyroiditis are significantly associated with prolonged OS in NSCLC patients treated with anti-PD-1 antibodies, even after accounting for ITB, in real-world clinical practice. We also confirmed that the occurrences of skin toxicity and overall irAEs were significantly associated with favorable outcomes, consistent with a previous retrospective study (10). Overall irAEs were significantly associated with prolonged OS because of the contribution of three irAEs (pituitary dysfunction, destructive thyroiditis, and skin toxicity) to improved OS. Attending physicians should be aware that patients with any of these three irAEs will have a better prognosis compared with patients without these irAEs.

Although many studies have explored the relationship between the development of irAEs and survival, few have evaluated the association between pituitary dysfunction and OS while taking into account ITB. One retrospective study demonstrated that the development of hypophysitis was associated with improved OS by univariate analysis, but this association disappeared after utilizing multivariate landmark survival analysis (14). Similarly, in another retrospective study, the survival benefit of pituitary dysfunction was not significant after accounting for ITB (15). One possible explanation for the discordant results between those two studies and ours is that pituitary dysfunction could have been overlooked, and the impact of pituitary dysfunction on OS might not have been accurately reflected in the previous retrospective studies. In fact, whereas 5.2% (10/194) of patients developed pituitary dysfunction after the initiation of anti-PD-1 antibodies in this study, only 1.1% (7/656) (14) and 1.2% (7/610) (15) of patients experienced pituitary dysfunction after anti-PD-1 antibodies in the other two studies, respectively.

Thyroid dysfunction is another frequent endocrine irAE. Most thyroid irAEs manifest as destructive thyroiditis with transient thyrotoxicosis, but they can also manifest as isolated hypothyroidism without preceding thyrotoxicosis (12, 16–21). In a previous retrospective study and a prospective study, thyroid dysfunction, including both destructive thyroiditis and isolated hypothyroidism, was correlated with longer OS after addressing ITB (10, 11). However, in our study, while the development of destructive thyroiditis was significantly associated with longer OS, isolated hypothyroidism and overall thyroid dysfunction were not. It is possible that the previous studies overlooked cases of isolated hypothyroidism, which develops significantly later compared with thyrotoxicosis (19). If this were the case, thyroid dysfunction in previous studies might have comprised mainly destructive thyroiditis. Consistent with our findings, Muir et al. reported in a retrospective study that the development of overt thyrotoxicosis, but not hypothyroidism, was associated with both prolonged OS and progression-free survival in patients with malignant melanoma, although their study did not take ITB into account (22). We recently reported greater changes in thyroid autoantibody (anti-thyroglobulin and anti-thyroid peroxidase antibodies) titers from baseline to irAE onset in patients with thyrotoxicosis than in those with isolated hypothyroidism (19). This finding suggests that the thyroid autoimmune response is greater in destructive thyroiditis than in isolated hypothyroidism. Therefore, it is possible that the anti-tumor immune response induced by ICIs may also be stronger in patients with destructive thyroiditis than in those with isolated hypothyroidism.

The reason why the impact on the outcomes varies among the different types of irAEs is still unknown. One hypothesis is that there may be common epitopes between irAE-associated antigens and tumor-associated antigens. Previous studies showed that the development of vitiligo as an irAE was associated with a favorable prognosis in melanoma patients (23). This is explained by activation of cytotoxic T lymphocytes targeting the common antigen between melanoma cells and normal melanocytes (24). Therefore, there might be shared antigens between pituitary or thyroid cells and NSCLC cells. Another possibility is that there might be some common germline genetic variants such as human leukocyte antigen (HLA) genotypes or single nucleotide polymorphisms related to both organ-specific irAEs and ICI efficacy. Recently, Jiang et al. reported that HLA-DRB4 was correlated with both the development of endocrine irAEs and improved OS in NSCLC patients receiving ICIs (25). In addition, the differences in survival impact depending on the type of irAE may be partially attributed to the differential management of irAEs according to the organ involved. ICI therapies are often discontinued and replaced with high-dose systemic corticosteroids in patients with severe non-endocrine irAEs such as hepatic toxicity, gastrointestinal toxicity, and pneumonitis (4, 26, 27). Several studies showed that high-dose corticosteroids can negatively affect the anti-tumor efficacy of ICIs (28, 29). Naqash et al. reported that ICI discontinuation after irAE development can also have a detrimental effect on OS (30). In contrast, patients with endocrine irAEs should not be treated with high-dose systemic corticosteroids and should continue ICIs under hormone replacement therapies according to most guidelines from academic societies (4, 13, 26, 27). Further studies are needed to understand the mechanism underlying the association between organ specific irAEs and ICI efficacy.

There are some limitations to this study. First, this was a single institutional study with a relatively small sample size. Second, it is possible that destructive thyroiditis occurred during the 6-week intervals between the thyroid function tests; the destructive thyroiditis might have gone undetected if the patient quickly progressed to hypothyroidism after preceding thyrotoxicosis. Third, while endocrine irAEs were evaluated prospectively as primary outcomes during scheduled endocrine evaluations during the follow-up period, non-endocrine irAEs were diagnosed clinically by the attending physicians and were not evaluated during the regularly scheduled evaluations in advance, which could lead to underestimation of their incidence. Fourth, the development of pituitary dysfunction may have been masked in patients treated with systemic glucocorticoids until the end of the study period.

## Conclusion

This prospective study clarified that the development of the specific irAEs (pituitary dysfunction, destructive thyroiditis, and skin toxicity) was associated with prolonged OS in NSCLC patients treated with anti-PD-1 antibodies even after accounting for ITB, suggesting that these irAEs could be prognostic biomarkers.

## Data Availability

The original contributions presented in the study are included in the article/**Supplementary Material**. Further inquiries can be directed to the corresponding authors.
