# Supplementary material for: Development of pituitary dysfunction and destructive thyroiditis is associated with better survival in non-small cell lung cancer patients treated with programmed cell death-1 inhibitors: a prospective study with immortal time bias correction
Source: Front Endocrinol (Lausanne). 2024 Nov 7;15:1490042. doi: 10.3389/fendo.2024.1490042 (PMC11578695; doi:10.3389/fendo.2024.1490042)
Supplement: Supplementary file 1 [file Table1.docx]

**Supplementary Material**

**Supplementary Table S1. Severity grade and management of each irAE type**

| IrAE | No. of patients | Grades 1–2 | Grades 3–5 | Systemic  steroid | Discontinuation of ICIs |
| --- | --- | --- | --- | --- | --- |
| Pituitary dysfunction | 11 | 0 (0%) | 11 (100%) | 0 (0%) | 4 (36.4%) |
| Thyroid dysfunction | 15 | 15 (100%) | 0 (0%) | 0 (0%) | 2 (13.3%) |
| Isolated hypothyroidism | 5 | 5 (100%) | 0 (0%) | 0 (0%) | 0 (0%) |
| Destructive thyroiditis | 10 | 10 (100%) | 0 (0%) | 0 (0%) | 2 (20.0%) |
| Skin toxicity | 27 | 23 (85.2%) | 4 (14.8%) | 3 (11.1%) | 7 (25.9%) |
| Pneumonitis | 24 | 16 (66.7%) | 8 (33.3%) | 18 (75.0%) | 19 (79.2%) |
| Hepatic toxicity | 11 | 5 (45.5%) | 6 (55.5%) | 5 (45.5%) | 6 (55.5%) |
| Gastrointestinal toxicity | 12 | 8 (66.7%) | 4 (33.3%) | 5 (41.7%) | 5 (41.7%) |

Abbreviations: irAE, immune-related adverse event; ICIs, immune checkpoint inhibitors.

**Supplementary Table S2. Baseline characteristics according to development of pituitary dysfunction**

| Characteristic | No. (%) or median (IQR) | | p value |
| --- | --- | --- | --- |
|  | With pit-irAE  (n = 11) | Without pit-irAE  (n = 183) |  |
| Age, years | 67 (72–75) | 69 (61–74) | 0.276 |
| <75 | 8 (72.7%) | 148 (80.9%) |  |
| ≥75 | 3 (27.3%) | 35 (19.1%) | 0.453 |
| Sex |  |  |  |
| Female | 3 (27.3%) | 45 (24.6%) |  |
| Male | 8 (72.7%) | 138 (75.4%) | 1.000 |
| ECOG-PS |  |  |  |
| ≤1 | 11 (100%) | 170 (92.9%) |  |
| ≥2 | 0 (0%) | 13 (7.1%) | 1.000 |
| Histology |  |  |  |
| Non-squamous | 8 (72.7%) | 127 (69.4%) |  |
| Squamous | 3 (27.3%) | 56 (30.6%) | 1.000 |
| Metastasis |  |  |  |
| No | 8 (72.7%) | 92 (50.3%) |  |
| Yes | 3 (27.3%) | 91 (49.7%) | 0.068 |

Abbreviations: IQR, interquartile range; pit-irAE, pituitary immune-related adverse event; ECOG-PS, Eastern Cooperative Oncology Group performance status.

**Supplementary Table S3. Baseline characteristics according to development of destructive thyroiditis**

| Characteristic | No. (%) or median (IQR) | | p value |
| --- | --- | --- | --- |
|  | With DT  (n = 10) | Without DT  (n = 184) |  |
| Age, years | 67 (58–72) | 70 (61–74) | 0.362 |
| <75 | 9 (90.0%) | 147 (79.9%) |  |
| ≥75 | 1 (10.0%) | 37 (20.1%) | 0.690 |
| Sex |  |  |  |
| Female | 4 (40.0%) | 44 (23.9%) |  |
| Male | 6 (60.0%) | 140 (76.1%) | 0.267 |
| ECOG-PS |  |  |  |
| ≤1 | 10 (100%) | 171 (92.9%) |  |
| ≥2 | 0 (0%) | 13 (7.1%) | 1.000 |
| Histology |  |  |  |
| Non-squamous | 7 (70.0%) | 128 (69.6%) |  |
| Squamous | 3 (30.0%) | 56 (30.4%) | 1.000 |
| Metastasis |  |  |  |
| No | 6 (60.0%) | 99 (53.8%) |  |
| Yes | 4 (40.0%) | 85 (46.2%) | 0.756 |

Abbreviations: IQR, interquartile range; DT, destructive thyroiditis; ECOG-PS, Eastern Cooperative Oncology Group performance status.

**Supplementary Table S4. Time-dependent multivariate Cox regression analysis of the association between overall irAEs on OS**

| Characteristic | Multivariate analysis | |
| --- | --- | --- |
|  | HR (95% CI) | p value |
| Age, years |  |  |
| <75 | 1 |  |
| ≥75 | 0.82 (0.52–1.29) | 0.390 |
| Sex |  |  |
| Female | 1 |  |
| Male | 1.06 (0.69–1.63) | 0.774 |
| ECOG-PS |  |  |
| ≤1 | 1 |  |
| ≥2 | 6.20 (3.24–11.87) | <0.001 |
| Histology |  |  |
| Non-squamous | 1 |  |
| Squamous | 1.13 (0.75–1.70) | 0.552 |
| Metastasis |  |  |
| No | 1 |  |
| Yes | 1.56 (1.10–2.21) | 0.013 |
| Overall irAEs |  |  |
| No | 1 |  |
| Yes | 0.14 (0.43–0.91) | 0.014 |

Abbreviations: irAE, immune-related adverse event; OS, overall survival; HR, hazard ratio; CI, confidence interval; ECOG-PS, Eastern Cooperative Oncology Group performance status.

**Supplementary Table S5. Time-dependent multivariate Cox regression analysis of the association between skin toxicity and OS**

|  | Multivariate analysis | |
| --- | --- | --- |
|  | HR (95% CI) | p value |
| Age, years |  |  |
| <75 | 1 |  |
| ≥75 | 0.77 (0.49–1.21) | 0.263 |
| Sex |  |  |
| Female | 1 |  |
| Male | 0.99 (0.64–1.52) | 0.954 |
| ECOG-PS |  |  |
| ≤1 | 1 |  |
| ≥2 | 6.76 (3.55–12.89) | <0.001 |
| Histology |  |  |
| Non-squamous | 1 |  |
| Squamous | 1.12 (0.75–1.69) | 0.577 |
| Metastasis |  |  |
| No | 1 |  |
| Yes | 1.59 (1.12–2.25) | 0.010 |
| Skin toxicity |  |  |
| No | 1 |  |
| Yes | 0.47 (0.25–0.85) | 0.013 |

Abbreviations: OS, overall survival; HR, hazard ratio; CI, confidence interval; ECOG-PS, Eastern Cooperative Oncology Group performance status.

**Supplementary Table S6. Baseline characteristics according to skin toxicity development**

| Characteristic | No. (%) or median (IQR) | | p value |
| --- | --- | --- | --- |
|  | With skin-irAE  (n = 27) | Without skin-irAE  (n = 167) |  |
| Age, years | 70 (62–73) | 70 (60–74) | 0.669 |
| <75 | 23 (85.2%) | 132 (79.0%) |  |
| ≥75 | 4 (14.8%) | 35 (21.0%) | 0.609 |
| Sex |  |  |  |
| Female | 9 (30.0%) | 39 (23.4%) |  |
| Male | 18 (70.0%) | 128 (76.6%) | 0.335 |
| ECOG-PS |  |  |  |
| ≤1 | 26 (96.3%) | 155 (92.8%) |  |
| ≥2 | 1 (3.7%) | 12 (7.2%) | 1.000 |
| Histology |  |  |  |
| Non-squamous | 22 (81.5%) | 113 (67.7％) |  |
| Squamous | 5 (18.5%) | 54 (32.3%) | 0.180 |
| Metastasis |  |  |  |
| No | 17 (63.0%) | 88 (52.7%) |  |
| Yes | 10 (37.0%) | 79 (47.3%) | 0.406 |

Abbreviations: IQR, interquartile range; irAE, immune-related adverse event; ECOG-PS, Eastern Cooperative Oncology Group performance status.

**Supplementary Table S7. Baseline characteristics according to overall irAE development**

| Characteristic | No. (%) or median (IQR) | | p value |
| --- | --- | --- | --- |
|  | With overall  irAEs (n = 82) | Without overall  irAEs (n = 112) |  |
| Age, years | 70 (61–74) | 70 (60–73) | 0.369 |
| <75 | 64 (78.0%) | 92 (82.1%) |  |
| ≥75 | 18 (22.0%) | 20 (17.9%) | 0.583 |
| Sex |  |  |  |
| Female | 20 (24.4%) | 28 (25.0%) |  |
| Male | 62 (75.6%) | 84 (75.0%) | 1.000 |
| ECOG-PS |  |  |  |
| ≤1 | 80 (97.6%) | 101 (90.2%) |  |
| ≥2 | 2 (2.4%) | 11 (9.8%) | 0.046 |
| Histology |  |  |  |
| Non-squamous | 59 (72.0%) | 76 (67.9%) |  |
| Squamous | 23 (28.0%) | 36 (32.1%) | 0.636 |
| Metastasis |  |  |  |
| No | 50 (71.0%) | 55 (49.1%) |  |
| Yes | 32 (39.0%) | 57 (50.9%) | 0.111 |

Abbreviations: irAE, immune-related adverse event; IQR, interquartile range; ECOG-PS, Eastern Cooperative Oncology Group performance status.
